# Tensile and Creep Testing of Sanicro 25 Using Miniature Specimens

**DOI:** 10.3390/ma11010142

**Published:** 2018-01-16

**Authors:** Petr Dymáček, Milan Jarý, Ferdinand Dobeš, Luboš Kloc

**Affiliations:** 1 Institute of Physics of Materials, Academy of Sciences of the Czech Republic, Žižkova 22, CZ-61662 Brno, Czech Republic; jary@ipm.cz (M.J.); dobes@ipm.cz (F.D.); kloc@ipm.cz (L.K.); 2 CEITEC IPM, Institute of Physics of Materials, Academy of Sciences of the Czech Republic, Žižkova 22, CZ-61662 Brno, Czech Republic

**Keywords:** Sanicro 25, austenitic steel, miniature tensile test, creep, fracture

## Abstract

Tensile and creep properties of new austenitic steel Sanicro 25 at room temperature and operating temperature 700 °C were investigated by testing on miniature specimens. The results were correlated with testing on conventional specimens. Very good agreement of results was obtained, namely in yield and ultimate strength, as well as short-term creep properties. Although the creep rupture time was found to be systematically shorter and creep ductility lower in the miniature test, the minimum creep rates were comparable. The analysis of the fracture surfaces revealed similar ductile fracture morphology for both specimen geometries. One exception was found in a small area near the miniature specimen edge that was cut by electro discharge machining, where an influence of the steel fracture behavior at elevated temperature was identified.

## 1. Introduction

Mechanical testing on miniature specimens is becoming increasingly important for several reasons. It can be used for testing irradiated materials to minimize the radiation dose, determining the local properties of weld zones or the remaining life of service exposed parts, the development of new materials available in limited amounts, etc. Different specimen geometries have been introduced in the past for tensile, fracture, and creep properties determination [1,2,3,4,5,6], including the well-known small punch test (SPT) and small punch creep test (SPC) [7,8,9,10,11,12,13,14,15,16,17,18,19,20]. The typical shape of the small punch specimen is most often a disc of 8 mm diameter or a square 10 × 10 mm and 0.5 mm thickness. The advantage of SPT or SPC is the need of higher forces for penetration of the disc (due to higher effective cross-section) in comparison to forces needed for testing a uniaxial miniature tensile specimen of cross-section in range of 0.5 to 2 mm^2^. A certain disadvantage of SPT and SPC is the equibiaxial stress state that makes correlation with uniaxial tests more complicated. The use of tensile specimens of similar size to an SP disc called a micro-tensile test (M-TT) was demonstrated in [21,22,23] with promising results. It has also been demonstrated that the flat specimens can represent the tensile curve well up to the necking point, and the ductility is influenced only by the post-necking region [1]. The use of a protective inert atmosphere is inevitable for testing miniature specimens at high temperatures to prevent specimen oxidation. The application of creep tests to miniature specimens is primarily intended for accelerated testing—mainly remaining life or local properties determination. It would hardly replace conventional long-term creep tests, and therefore a reasonable length of such an experiment is within the 1000 h range.

The aim of this study is to obtain tensile and creep properties using miniature tensile specimens prepared from the same *D*_s_ = 8 mm, similar to the SPT disc size, and correlate the results with measurements on standard specimens for the prospective austenitic steel Sanicro 25. This should demonstrate the potential of miniature specimen testing methods to investigate the mechanical properties of new materials at room and elevated temperatures.

## 2. Materials and Methods

**Figure 1:**
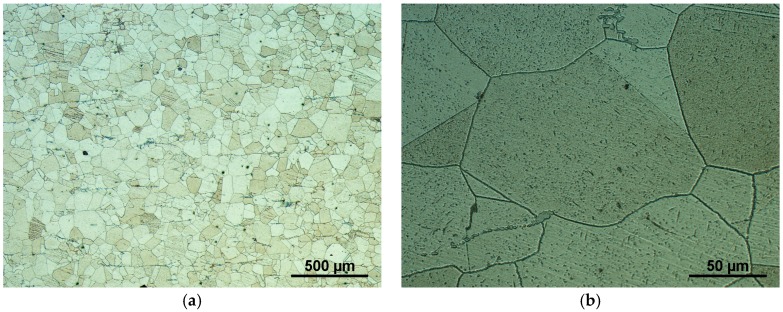
Sanicro 25 austenitic microstructure, etched in hydrochloric acid and hydrogen peroxide water solution, magnification (**a**) 50× and (**b**) 500×.

Austenitic steel of grade UNS S31035, Sanicro 25 [24], was selected as an experimental material for the study. It was produced by Sandvik (Sandviken, Sweden) in the form of a seamless tube of 38 mm in diameter and wall thickness of 8.8 mm, heat no. 527207, and lot no. 24476. It was supplied to Doosan Babcock Energy Ltd. (Renfrew, UK) and part of the tube was provided to Institute of Physics of Materials, ASCR (IPM) for creep testing within the European Virtual Institute on Knowledge-based Multifunctional Materials (KMM-VIN) research activities [25]. The chemical composition is shown in Table 1. The experimental material was used in the as-received state after solution annealing 1220 °C/5 min/cooled in water. A fine twinned austenitic microstructure of the steel is shown in Figure 1a. It contains grains with average size of about 25 μm, but grains with size of up to 200 μm are also present in the microstructure, as shown in Figure 1b. The grain boundaries are decorated with Nb-rich carbonitrides of typical size 200 nm, as reported in [26,27]. The carbonitrides are also present in the interior of larger grains, and their chemistry is also discussed in [26,27]. Sanicro 25 shows very good creep and fatigue resistance at high temperatures up to 700 °C, as well as oxidation resistance [24,28]. Significant strain hardening and cyclic strain hardening of the steel has been demonstrated due to interaction of the dislocations with about 50-nm nanoclusters at high temperatures [24,29].

All creep tests were performed in purified Ar 4.6 atmosphere using a lever arm (10:1) 8 kN creep machine of IPM design for testing standard specimens. The machine was additionally equipped with special grips designed for miniature specimens. The elongation was recorded via linear variable differential transformer (LVDT), and the force was monitored by a 10 kN load cell located on the bottom side of the load train. The LVDT is located below the load train of the creep machine and it measures the specimen elongation indirectly from the movement of the upper grip using two transmitting pullrods. The machine was equipped with a stepping motor connected with the deadweight table to enable constant rate experiments. In this case, a deadweight of the full capacity of the machine (80 kg) was applied. The loading rate 0.25 mm/min was used for miniature tensile tests. Uniaxial tensile tests on standard specimens were performed in a 50 kN electromechanical creep machine (Messphysik KAPPA 50 LA-Spring, Fürstenfeld, Austria) with Maytec furnace in Ar 5.0 atmosphere at strain rate 10^−3^ s^−1^. In this case a Maytec high-temperature extensometer (Singen, Germany) was used to measure the elongation of the specimen’s cylindrical gauge length.

**Figure 2:**
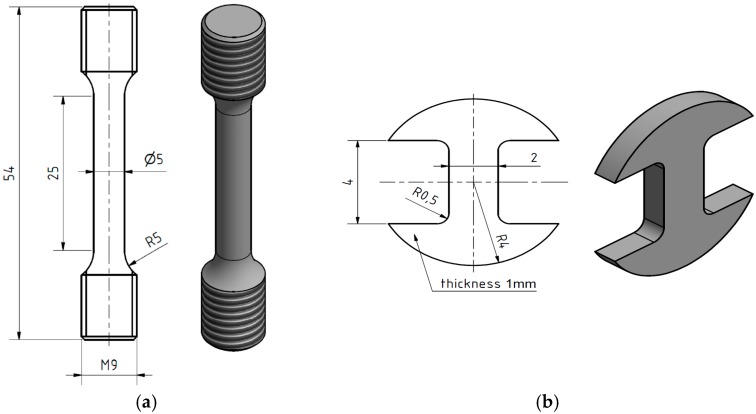
(**a**) Standard and (**b**) miniature tensile specimen.

The standard specimen is shown in Figure 2a. Miniature specimens were 8 mm in diameter and 1 mm ± 0.005 mm thick. They were prepared from a cylinder by electro discharge machining (EDM) to 1.2 mm-thick slices and ground from both sides to final thickness under water on metallographic papers up to 2500 grit. After this, they were cut by EDM to the required shape according to Figure 2b. 

The tensile tests as well as creep tests were performed one time per test condition. A larger set of specimens for testing repeatability or statistical treatment was not at our disposal. The microstructure of the studied steel was well homogenous and the consistency of the tensile and creep results presented in following indicates that the scatter should be at a low level.

## 3. Results and Discussion

### 3.1. Tensile Properties

**Figure 3:**
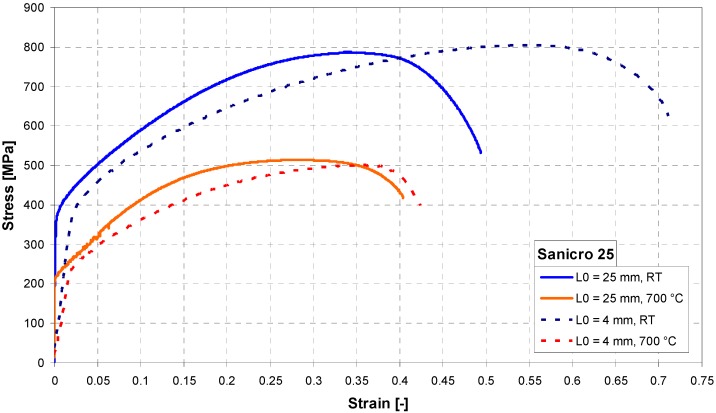
Tensile curves of Sanicro 25 steel.

**Figure 4:**
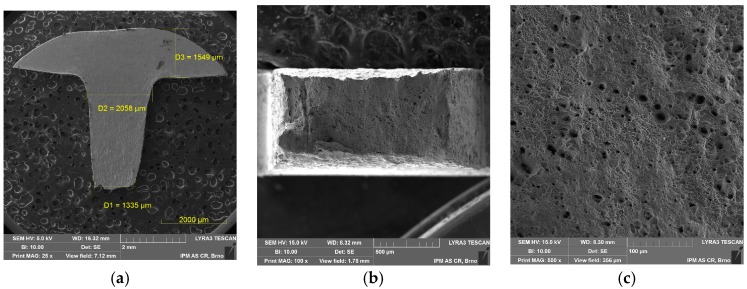
SEM fractographs of miniature tensile specimen tested at room temperature (RT): (**a**) magnification 25×; (**b**) specimen fracture 100×; (**c**) specimen center 500×.

**Figure 5:**
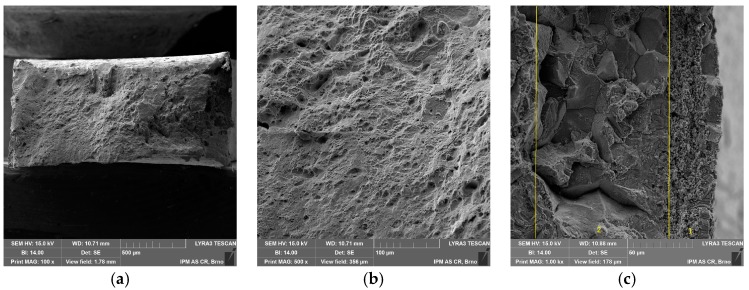
SEM fractographs of miniature tensile specimen tested at 700 °C: (**a**) specimen fracture 100×; (**b**) specimen center 500×; (**c**) specimen right side 1000×.

Tensile properties at room temperature (RT) and elevated temperature (700 °C) were measured using both specimen geometries. As shown in Figure 3, the tensile curves differ mainly due to the method of deformation measurement (extensometer on specimen vs. extensometer on grips). This is most evident in the slope of the elastic part of the curves. The miniature specimen had a relatively small head and it visibly plastically deformed (pulled in) during the tensile test at room temperature, as shown in Figure 4a. Such a deformation of the specimen head was not found at 700 °C. Fracture surfaces of both specimens tested at RT and 700 °C showed clear signs of ductile transgranular fracture. Dimples were present in most of the fracture surface, as shown in Figure 4b,c (test at RT) and Figure 5a,b (test at 700 °C). However, signs of an intergranular fracture were also found as shown in Figure 5c, in a small location near the specimen side, which was machined by EDM. Different fracture morphology in zone 1 of about 40 μm depth is visible in Figure 5c, which is generally considered as the zone influenced by high energy accumulation by EDM, but another zone 2 at about 80–100 μm deep clearly shows intergranular fracture morphology. This could point to a higher sensitivity of Sanicro 25 to EDM than other steels, and should be studied further. 

Despite these observations, the agreement of ultimate tensile and yield strength values obtained is very good, as shown in Table 2. The ultimate tensile strength obtained was practically identical. The yield strength was identical at room temperature and less than 10% higher at 700 °C. The maximum forces *F*_m_ obtained in both types of tensile tests are shown in Table 3. The ratio of *F*_m_ for standard/miniature specimen was ~10, which is approximately the ratio of the two specimen type cross-sections (19.635 mm^2^/2 mm^2^ = 9.82).

A further detailed optimization of SPT sized miniature tensile specimen geometry, especially its gauge length, cross-section, and head size, would be beneficial by finite element method and experimentally. The M-TT specimen geometry described in [21,22,23] seems preferable for static testing in order to avoid the excessive plastic deformation of the specimen head. Broad discussion is needed to initiate a process that would lead to the standardization of this miniature test.

### 3.2. Creep

**Figure 6:**
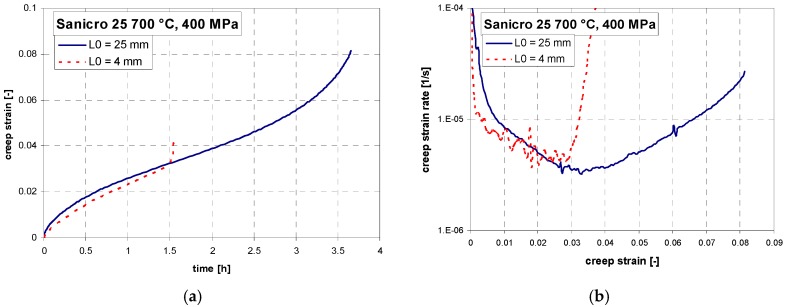
(**a**) Creep curves; (**b**) creep rate vs. strain relations of Sanicro 25 at 700 °C, 400 MPa.

**Figure 7:**
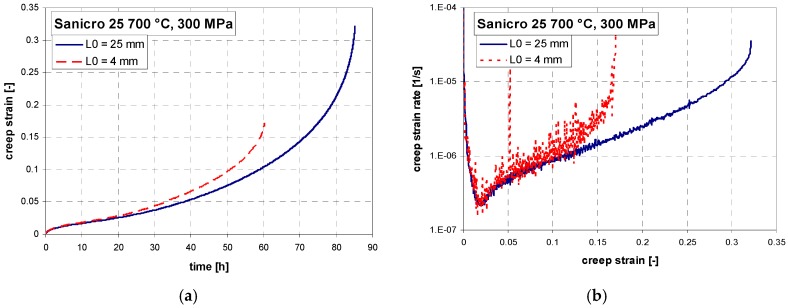
(**a**) Creep curves; (**b**) creep rate vs. strain relations of Sanicro 25 at 700 °C, 300 MPa.

**Figure 8:**
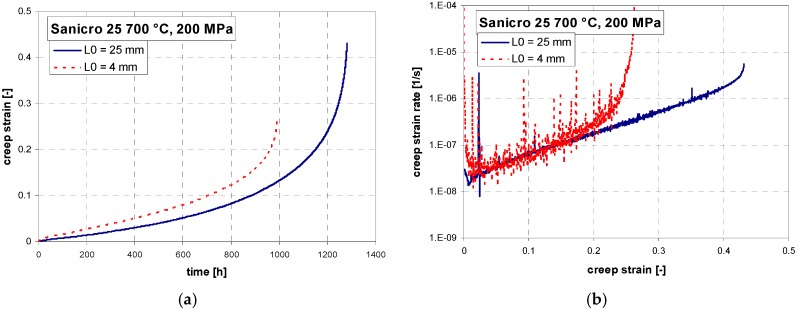
(**a**) Creep curves; (**b**) creep rate vs. strain relations of Sanicro 25 at 700 °C, 200 MPa.

**Figure 9:**
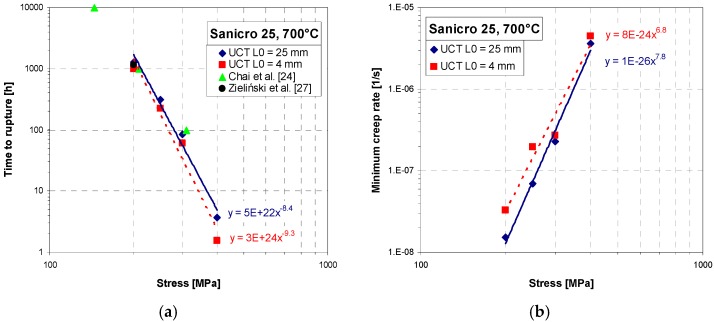
(**a**) Time to rupture dependence on stress; (**b**) minimum creep rate dependence on stress.

**Figure 10:**
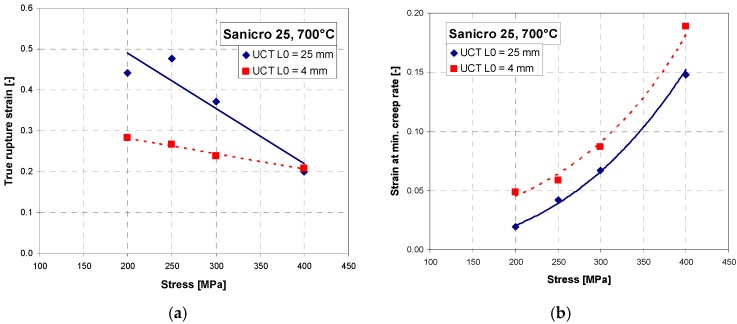
(**a**) True rupture stress dependence on applied stress; (**b**) strain at min. creep rate dependence on applied stress.

Short-term creep testing was performed on both specimen geometries at 700 °C. The creep curves and strain rate dependence on creep strain for stress between 200 to 400 MPa are shown in Figure 6, Figure 7 and Figure 8. Except for the test at 200 MPa, all of the tests were conducted above the yield strength of the steel. The initial strain obtained instantly after loading was subtracted, so only creep strain is plotted in the creep curves. It can be seen from Figure 6a, Figure 7a, Figure 8a and Figure 9a that for all tests the time to rupture was lower for miniature specimens. Figure 9a also shows that the time to rupture at 200 MPa is well comparable with results obtained in [24] and [27]. The creep strain rate (derivative of creep strain) is shown in Figure 6b, Figure 7b and Figure 8b. There was a higher noise for miniature specimens compared to standard specimens. This is attributed to the specimen length and given sensitivity and noise of the induction sensor recording the deformation. The stress exponent *n* is approximately 8 for standard specimens and 7 for miniature specimens, as shown in Figure 9b. The rupture strain dependence on stress is plotted in Figure 10a. It is shown that the rupture strain steadily increases with decreasing stress. For miniature specimens, it increased less than for standard specimens. This can be attributed to small gauge length. The strain at minimum creep rate dependence on stress is plotted in Figure 10b. It is systematically higher for miniature specimens compared to standard specimens. Creep rupture time of the miniature specimens was in all cases approximately 30–40% lower compared with standard specimens, and the difference slightly decreased with lower stress. This represents a certain level of conservative factor if the miniature specimen testing method is applied instead of the standard test. 

**Figure 11:**
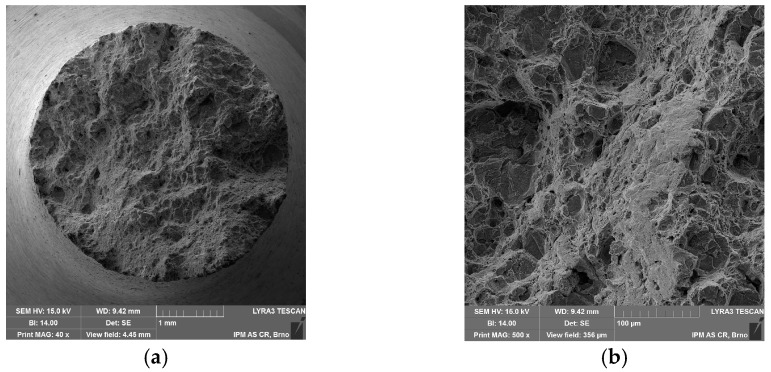
SEM fractographs of standard creep specimen (σ = 300 MPa, *T* = 700 °C, *t*_r_ = 85.2 h): (**a**) fracture surface 40×; (**b**) fracture surface 500×.

**Figure 12:**
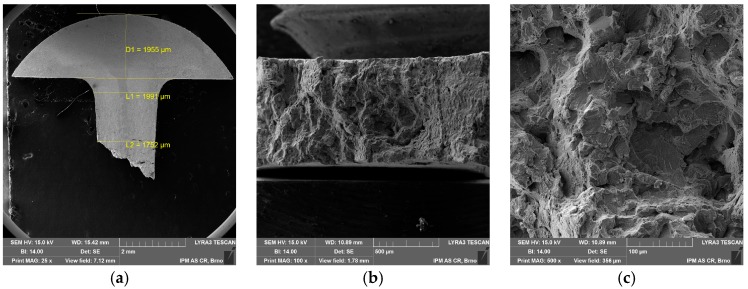
SEM fractographs of miniature creep specimen (σ = 300 MPa, *T* = 700 °C, *t*_r_ = 60.2 h): (**a**) magnification 25×; (**b**) fracture surface 40×; (**c**) fracture surface 500×.

The fractographic analysis does not show any significant differences between the standard and miniature creep specimen fracture. Fracture morphology is mainly ductile transgranular, as shown in Figure 11 and Figure 12. There is the presence of dimples and quasi-cleavage fracture that can also be found in other austenitic steels (e.g., 316L) [15]. However, the presence of dimples is significantly decreased in creep fracture (Figure 11b and Figure 12c) in comparison with tensile test fracture at high temperature (Figure 5b). The miniature specimen head visibly did not deform in the creep test as in the case of the tensile test at room temperature (Figure 12a compared to Figure 4a).

### 3.3. Perspective of Miniature Specimen Testing

The authors believe that the results presented herein are promising. Based on these results, research should further proceed to broader studies that concentrate on: (i) optimization of specimen dimensions; (ii) testing various materials, such as ferritic-martensitic steels, austenitic steels, light alloys, and others in a similar way as SPT and SPC [8,9,10,11,12,13,14,15,16,17,18,19,20]; and (iii) application to practical tasks for the industry (e.g., determination of weld properties or service life extension in a similar manner as presented in [27,30,31]). It is necessary to define the size effect related to the particular microstructures and specimen geometry. Additionally, it is key to prove the repeatability and reproducibility of the test results on different testing machines. After successful resolution of these challenging tasks, the standardization of the miniature tensile and creep test should be feasible.

## 4. Conclusions

Conventional and miniature tensile and creep tests were applied to identify the mechanical properties of the new austenitic steel Sanicro 25 at room and elevated temperatures. Based on the comparison of both types of test results, it is possible to draw following conclusions:

- Miniature specimens can give very precise estimation of mechanical properties from a very small volume of material
- The creep life of miniature specimens was about 30–40% lower than that of standard ones, and the difference decreased with lower stress, mainly due to lower ductility
- Comparable minimum creep rates at the same stress were obtained from both types of tests
- Special care must be paid if the miniature specimens are prepared by different technology than the standard specimens (here machining and grinding vs. grinding and EDM), since changes in the fracture behavior of the steel at elevated temperature were demonstrated in a small local area.

## Figures and Tables

**Table 1:** Chemical composition of Sanicro 25.

**Element**	**C**	**Ni**	**Cr**	**W**	**Co**	**Cu**	**Mn**
wt %	0.064	25.36	22.35	3.37	1.44	2.98	0.51
**Element**	**Nb**	**N**	**Si**	**N**	**P**	**B**	**Fe**
wt %	0.49	0.23	0.18	0.23	0.016	0.0035	balance

**Table 2:** Results of uniaxial tensile tests at RT and 700 °C.

Method/Condition	Ultimate Tensile Strength *R*_m_ (MPa)		Proof Yield Strength *R*_p0.2_ (MPa)	
Temperature	RT	700 °C	RT	700 °C
Miniature tensile test	804	500	376	238
Standard tensile test	787	514	375	217
Inspection certificate tensile test	786	514	369	202
Difference miniature vs. standard	+2.2%	−2.7%	+0.3%	+9.7%

**Table 3:** Maximum forces *F*_m_ corresponding to *R*_m_ in the uniaxial tensile tests at RT and 700 °C.

Method/Condition	Maximum Force *F*_m_ (N)	
Temperature	RT	700 °C
Miniature tensile test	1551	955
Standard tensile test	15,450	10,086
Ratio *F*_m_ standard/*F*_m_ miniature	9.96	10.56
